# Cytotoxic Evaluation of (2*S*)-5,7-Dihydroxy-6-prenylflavanone Derivatives Loaded PLGA Nanoparticles against MiaPaCa-2 Cells

**DOI:** 10.3390/molecules22091553

**Published:** 2017-09-15

**Authors:** Berenice Andrade-Carrera, Beatriz Clares, Véronique Noé, Mireia Mallandrich, Ana C. Calpena, María Luisa García, María Luisa Garduño-Ramírez

**Affiliations:** 1 Centro de Investigaciones Químicas, Instituto de Investigación en Ciencias Básicas y Aplicadas, Universidad Autónoma del Estado de Morelos, Avenida Universidad 1001, Col. Chamilpa, Cuernavaca, Morelos 62209, Mexico; bereniceac@uaem.mx; 2 Department of Pharmacy and Pharmaceutical Technology, School of Pharmacy, Campus of Cartuja s/n, University of Granada, 18071 Granada, Spain; beatrizclares@ugr.es; 3 Nanoscience and Nanotechnology Institute (IN2UB), University of Barcelona, 27-31 Joan XXIII Avenue, 08028 Barcelona, Spain; mireia.mallandrich@ub.edu (M.M.); anacalpena@ub.edu (A.C.C.); marisagarcia@ub.edu (M.L.G.); 4 Department of Biochemistry and Physiology, Faculty of Pharmacy and Food Science, University of Barcelona, 27-31 Joan XXIII Avenue, 08028 Barcelona, Spain; vnoe@ub.edu; 5 Department of Pharmacy, Pharmaceutical Technology and Physical Chemistry, School of Pharmacy and Food Sciences, University of Barcelona, 27-31 Joan XXIII Avenue, 08028 Barcelona, Spain

**Keywords:** flavanone, *Eysenhardtia*, cytotoxic activity, MiaPaCa-2

## Abstract

The search for new alternatives for the prevention and treatment of cancer is extremely important to minimize human mortality. Natural products are an alternative to chemical drugs, since they are a source of many potential compounds with anticancer properties. In the present study, the (2*S*)-5,7-dihydroxy-6-prenylflavanone (semi-systematic name), also called (2*S*)-5,7-dihydroxy-6-(3-methyl-2-buten-1-yl)-2-phenyl-2,3-dihydro-4*H*-1-Benzopyran-4-one (CAS Name registered) (**1**) was isolated from *Eysenhardtia platycarpa* leaves. This flavanone **1** was considered as the lead compound to generate new cytotoxic derivatives **1a**, **1b**, **1c** and **1d**. These compounds **1**, **1a**, **1b**, **1c**, and **1d** were then loaded in nanosized drug delivery systems such as polymeric nanoparticles (NPs). Small homogeneous spherical shaped NPs were obtained. Cytotoxic activity of free compounds **1**, **1a**, **1b**, **1c**, and **1d** and encapsulated in polymeric NPs (NPs**1**, NPs**1a**, NPs**1b**, NPs**1c** and NPs**1d**) were evaluated against the pancreatic cancer cell line MiaPaCa-2. The obtained results demonstrated that NPs**1a** and NPs**1b** exhibited optimal cytotoxicity, and an even higher improvement of the cytotoxic efficacy was exhibited with the encapsulation of **1a**. Based on these results, NPs**1a** were proposed as promising anticancer agent candidates.

## 1. Introduction

Flavonoids are low molecular weight polyphenolic compounds derived from secondary metabolism of plants. The structural substitution in the flavonoid ring system with prenyl groups increases the lipophilicity and its affinity for biological membranes [1]. Several prenylated flavanones with antioxidant, anti-inflammatory, hepatoprotective and/or cytotoxic activities have been widely identified from natural sources [2]. Concretely, flavanones obtained from *Fabaceae* family have been reported to be compounds with some functional groups granting them higher biological activity [3], such as chalcones, isoflavones and flavanones with functionalized groups at specific positions [4], as well as flavanones linked to pyran ring [5]. These functional group modifications have been found to increase their pharmacological effects, mainly the anti-inflammatory and cytotoxic actions [6].

In a previous work the anti-inflammatory, antioxidant and cytotoxic activities of natural flavanones isolated from the methanolic extract of *Eysenhardtia platycarpa* leaves have been reported [2]. Flavanones exhibited anti-inflammatory activity in an inflammatory induction model on mouse ear and cytotoxic activity by the *Artemia salina* (brine shrimp) method. Different structural modifications such as esterification (compound **1a**), methylation (compound **1b**), cyclization (compound **1c**) or vinylogous cyclization (compound **1d**) of the isolated flavanone **1** represent a suitable strategy to obtain new compounds with improved biological efficacy as wells as a strategy to evaluate the influence of the substitution pattern on the structure–activity relationship.

Among the broad spectrum of biological activities of flavonoids in general and concretely, in flavonoids with prenylated phenol groups, cytotoxicity is one of the most investigated pharmacological effects [7]. In this context the search of new or improved anti-cancer molecules is a challenging field for scientists. The main goal of cancer therapy is to kill the tumor cells, and the initial step in the evaluation of the potential antitumor efficacy of new bioactive substances is the assessment of their cytotoxic effect in human cancer cell lines.

The vehiculization of the active substance in an appropriate delivery system (liposomes, solid lipid nanoparticles, nanoemulsios and polymeric nanoparticles, among others) provides advantages and therapeutic benefits in term of kinetics release delivery, biodistribution regulation and minimization of significant side effect. These benefits have been observed in natural flavanones loaded in nanoemulsion and poly(dl-lactide-*co*-glycolide acid) (PLGA) nanoparticles (NPs) [8,9].

In this case, PLGA-NPs were considered as suitable delivery systems due to their ability for enhancing the bioavailability of hydrophobic drugs such as the natural flavanone **1** and derivatives compounds **1a**, **1b**, **1c** and **1d** which possess hydrophobic nature given by their functional groups based on one of their functional components [10].

Among the wide range of nanosystems for drug delivery, NPs play an important role in the cancer treatment providing additional advantages over the use of free drugs [11]. Among them, PLGA is one of the best reported polymers for the elaboration of biomedical NPs due to its biocompatibility. PLGA is easily metabolized into lactic and glycolic acids and excreted by the body as carbon dioxide and water [12].

**Figure 1:**
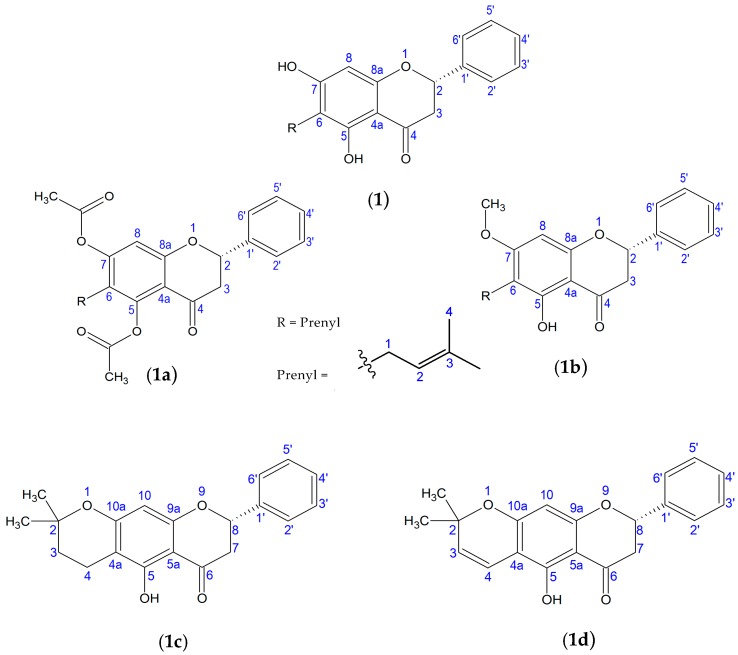
Chemical structures of the studied compounds. (2*S*)-5,7-dihydroxy-6-(3-methyl-2-buten-1-yl)-2-phenyl-2,3-dihydro-4*H*-1-Benzopyran-4-one (**1**); (2*S*)-5,7-bis(acetyloxy)-6-(3-methyl-2-buten-1-yl)-2-phenyl-2,3-dihydro-4*H*-1-Benzopyran-4-one (**1a**); (2*S*)-5-hydroxy-7-methoxy-6-(3-mehyl-2-buten-1-yl)-2-phenyl-2,3-dihydro-4*H*-1-Benzopyran-4-one (**1b**); (8*S*)-5-hydroxy-2,2-dimethyl-8-prenyl-3,4,7,8-tetrahydro-2*H*,6*H*-Benzo[1,2-*b*:5,4-*b*′]dipyran-6-one (**1c**) and (8S)-5-hydroxy-2,2-dimethyl-8-phenyl-7,8-dihydro-2*H*,6*H*-Benzo[1,2-*b*:5,4-*b*′]dipyran-6-one (**1d**).

Based on the previous comments and considering the potential cytotoxicity of flavanones isolated from *E. platycarpa* leaves: (2*S*)-5,7-dihydroxy-6-(3-methyl-2-buten-1-yl)-2-phenyl-2,3-dihydro-4*H*-1-Benzopyran-4-one (**1**), as well as derivatives obtained from structural modifications: (2*S*)-5,7-bis(acetyloxy)-6-(3-methyl-2-buten-1-yl)-2-phenyl-2,3-dihydro-4*H*-1-Benzopyran-4-one (**1a**); (2*S*)-5-hydroxy-7-methoxy-6-(3-mehyl-2-buten-1-yl)-2-phenyl-2,3-dihydro-4*H*-1-Benzopyran-4-one (**1b**); (8*S*)-5-hydroxy-2,2-dimethyl-8-prenyl-3,4,7,8-tetrahydro-2*H*,6*H*-Benzo[1,2-*b*:5,4-*b*′]dipyran-6-one (1c); and (8S)-5-hydroxy-2,2-dimethyl-8-phenyl-7,8-dihydro-2*H*,6*H*-Benzo[1,2-*b*:5,4-*b*′]dipyran-6-one (**1d**) (Figure 1) might be considered as optimal candidates for the investigation as anticancer agents. The main aim of this study was the evaluation of the cytotoxic activity of these flavonoids compounds **1**, **1a**, **1b**, **1c** and **1d** free and loaded in effective PLGA-NPs against pancreatic cancer cell line (MiaPaCa-2).

## 2. Results and Discussion

### 2.1. Chemical Characterization

The structural elucidation of the flavanone **1** was reported by Ahluwalia et al. [13] and the (2*S*) configuration structure by Narvaez et al. [14]. Flavanone **1** (Figure 1) was obtained (0.329 g) as a yellow solid with a melting point of 200–202 °C. It showed a molecular ion of *m*/*z* 324 in the mass spectrum, in addition to the peaks of typical fragmentations of previously described flavanones [15]. The molecular ion also confirmed the molecular formula C_20_H_20_O_4_. The infrared (IR) spectrum showed bands for hydroxyl group in 3134 cm^−1^ and a band in 1634 cm^−1^ corresponding to a carbonyl group. Characterization was also performed by spectroscopic techniques such as: ^1^H nuclear magnetic resonance (NMR), ^13^C-NMR, correlated spectroscopy (COSY), distortion-less enhancement by polarization transfer (DEPT), heteronuclear single quantum correlation spectroscopy (HSQC), (heteronuclear multiple bond correlation) HMBC and nuclear overhauser enhancement spectroscopy (NOESY).

### 2.2. Pharmacomodulation

Pharmacomodulation is a molecular design strategy in which an already established lead compound is modified to improve its pharmacokinetic and/or pharmacodynamics parameters. Flavanone **1** (as lead compound) was considered in the present work for the development of new active principles as new therapeutic agents with enhanced efficacy and bioavailability, and reduced toxicity and undesired side effects [16].

#### 2.2.1. Esterification

**Scheme 1:**
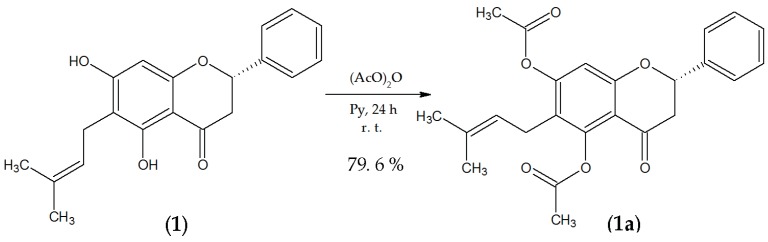
Esterification reaction of (2*S*)-5,7-dihydroxy-6-(3-methyl-2-buten-1-yl)-2-phenyl-2,3-dihydro-4*H*-1-Benzopyran-4-one (**1**) to obtain (2*S*)-5,7-bis(acetyloxy)-6-(3-methyl-2-buten-1-yl)-2-phenyl-2,3-dihydro-4*H*-1-Benzopyran-4-one (**1a**).

Esterification was one of the strategies to study the pharmacomodulation of the lead compound flavanone **1**. The ester derivative **1a** allows one to increase the lipophilicity, which confers greater capacity for the transport through cell membranes [16]. In many cases, the active substance needs to reach the cytosol to exert its biological action. This could be an essential condition in the case of some anticancer agents depending on their mechanisms of action. For this reason exogenous molecules must first traverse the cell membrane. If the membrane transport is addressed by passive diffusion the minimizing of the effective polarity of the desired compound maybe, improve its permeation properties by esterification strategy shielding polar groups [17]. The esterification was performed with acetic anhydride over pyridine to obtain the esterified derivate **1a** (Scheme 1).

**Scheme 2:**
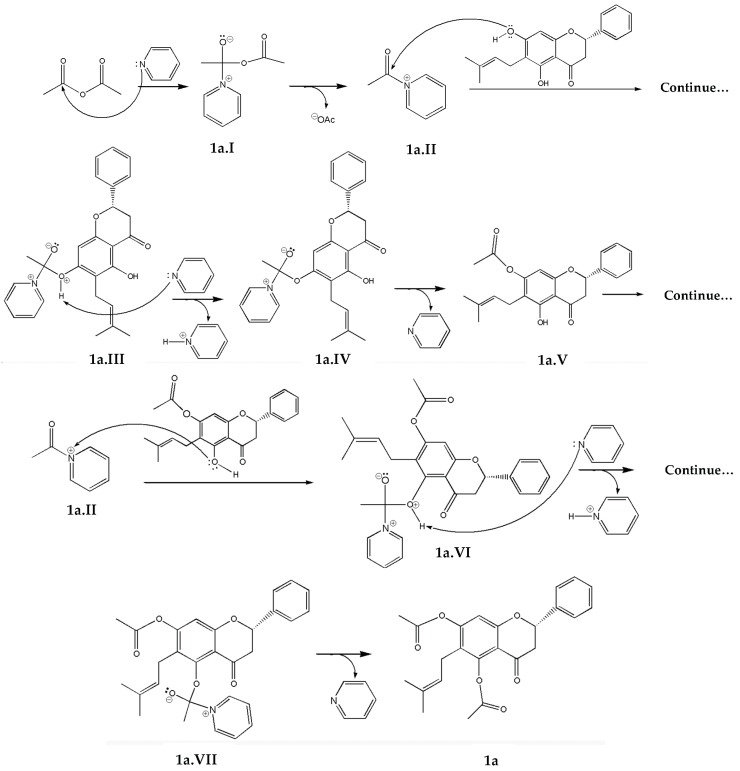
A plausible mechanism for the semi-synthesis of (2*S*)-5,7-bis(acetyloxy)-6-(3-methyl-2-buten-1-yl)-2-phenyl-2,3-dihydro-4*H*-1-Benzopyran-4-one (**1a**).

The proposed reaction mechanism for this reactions is shown in Scheme 2, where acetic anhydride was used as the acylating agent, which is attacked by the pair of electrons of pyridine to give the intermediate switterion **1a.I**, which by loss of an acetate ion leads to intermediate **1a.II**, which in turn is attacked by the pair of electrons of the oxygen of the hydroxyl group from position 6 of flavanone **1** to afford intermediate **1a.III**, which by abstraction of the proton forms the intermediate **1a.IV** plus a protonated pyridine, which is subsequently neutralized by the acetate anion regenerating the neutral pyridine and acetic acid to form the intermediate **1a.V**, which corresponds to a mono-acetylated compound. The next stage of the reaction consists of the attack of the electron pair of intermediate **1a.V** on the second molecule of intermediate **1a.II** resulting the intermediate **1a.VI**, in which by abstraction of the proton bounds to oxygen by a new molecule of pyridine leads to the formation of intermediate **1a.VII** plus the protonated pyridine, which is subsequently neutralized with a second acetate anion to finalize the total reaction process of the product (**1a**).

Flavanone **1a** was obtained as a yellow solid substance with a melting point of 72–78 °C, which showed a [M + 1]^+^ molecular ion peak at *m*/*z* 409.1853 in mass spectrum obtained in ESI ion positive mode and characteristic bands in the IR spectrum for ketone carbonyl to C-4, ester carbonyl at C-5 and C-7, as well as double bond in prenyl moety and aromatic rings (rings B and C of flavanone).

Analyzing the ^1^H-NMR spectrum was possible to observe the flavanone characteristics signals [14] and typical signals for an acetate group at 2.32 and 2.43 ppm. In the ^13^C-NMR spectrum the presence of two signals at 168.02 and 169.05 ppm belonging to the carbonyl of two ester groups were also observed.

Other carbon and hydrogen peak assignments were performed through comparison with the signals obtained from natural flavanone **1**. Furthermore the 2D correlation spectrum ^1^H-^13^C, HSQC and NOESY confirmed the identity of the obtained modulated ester **1a**. This compound is considered novel because was not reported previously in the literature.

#### 2.2.2. Methylation

Methylation is another strategy to modulate the interaction with biological membranes. Methylated flavanones are less polar, a thus display enhanced metabolic stability and membrane transport properties, leading to improved absorption and greatly increased oral bioavailability [18]. An increased cancer chemoprotective effect has been reported in flavones after methylation [19], however, the main disadvantage of this strategy is that aqueous solubility decrease.

**Scheme 3:**
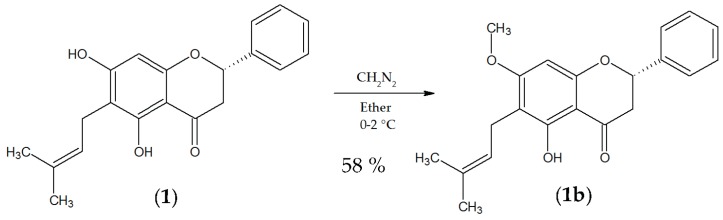
Methylation reaction of (2*S*)-5,7-dihydroxy-6-(3-methyl-2-buten-1-yl)-2-phenyl-2,3-dihydro-4*H*-1-Benzopyran-4-one (**1**) to produce (2*S*)-5-hydroxy-7-methoxy-6-(3-mehyl-2-buten-1-yl)-2-phenyl-2,3-dihydro-4*H*-1-Benzopyran-4-one (**1b**).

Diazomethane was used as the alkylating agent for the methylation reaction to obtain the corresponding modulated ether **1b** (Scheme 3) [20]. The polarity of the natural flavanone **1** was thus modified by forming a methylated derivative, which lipophilicity got better for transport through biological membrane [16].

**Scheme 4:**
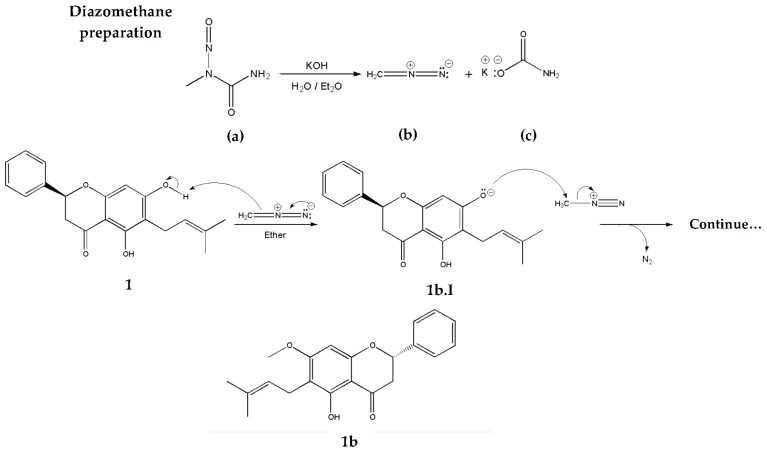
Proposed mechanism for semi-synthesis of (2*S*)-5-hydroxy-7-methoxy-6-(3-mehyl-2-buten-1-yl)-2-phenyl-2,3-dihydro-4*H*-1-Benzopyran-4-one (**1b**).

Diazomentane (**b**) is prepared by hydrolysis of an ethereal solution of *N*-nitroso-*N*-methylurea (**a**) with potassium hydroxide, obtaining methyl diazotate (**c**) as a subproduct as depicted in Scheme 4. Once diazomethane (**b**) was prepared, it was reacted with flavanone **1** and diazomethane abstracted the hydroxyl proton from the 7-position alcohol of flavanone **1** to form the alkoxide in intermediate **1b.I**, which subsequently abstracted the methyl from the methyl carbamate releasing molecular nitrogen and resulting in the product **1b**.

The alkyl modulated compound **1b** was a yellow solid, with melting point of 95–98 °C and molecular formula C_21_H_22_O_4_. The IR spectrum exhibited the characteristic bands corresponding to vibration hydroxyl groups, carbonyl ketone double bond and aromatic rings. The ^1^H-NMR spectrum of **1b** was similar to that of **1** with the exception of a singlet signal at 3.83 ppm corresponding to the methyl ether, now present in the C-7 position and matching the methyl ether carbon signal at 56.14 ppm in the ^13^C-NMR spectrum. Likewise, this was corroborated by heteronuclear correlation observed in its ^1^H-^13^C-HSQC spectrum; the structural elucidation was confirmed by data reported by Filho et al. [21].

#### 2.2.3. Cyclization

Within the pharmacomodulation techniques, ring formation is an approach for the study of the active conformation in the starting molecule (lead compound). Since the product is a molecule with less conformational freedom, one of the most frequent drawbacks of this modification is that the introduction of structural elements might modify both the activity and the physicochemical properties of the natural flavanone **1**. On the other hand, the formation of rings can lead to the creation of new stereogenic centers, with all the repercussions that this entails in terms of racemic mixtures and enantiomeric excesses among others. This cyclization strategy could have both structure—metabolism and structure–toxicity relationship implications. It has been reported to improve the in vitro anticancer activity against human cancer cell lines [22] of flavanones lead compounds.

**Scheme 5:**
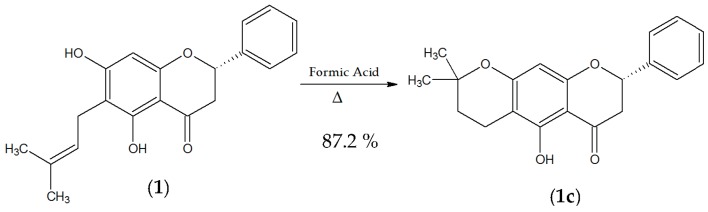
Cyclization reaction of (2*S*)-5,7-dihydroxy-6-(3-methyl-2-buten-1-yl)-2-phenyl-2,3-dihydro-4*H*-1-Benzopyran-4-one (**1**) to form (8*S*)-5-hydroxy-2,2-dimethyl-8-prenyl-3,4,7,8-tetrahydro-2*H*,6*H*-Benzo[1,2-*b*:5,4-*b*′]dipyran-6-one (**1c**).

The reaction conditions for obtaining modulated compound **1c** (Scheme 5) were described by Jain et al. [23], for carrying out the cyclization strategy between the hydroxyl group in position 7 and prenyl group in position 6 at flavanone **1** to form a pyran **1c**. This kind of synthesis strategy was also described for other flavonoids [13].

**Scheme 6:**
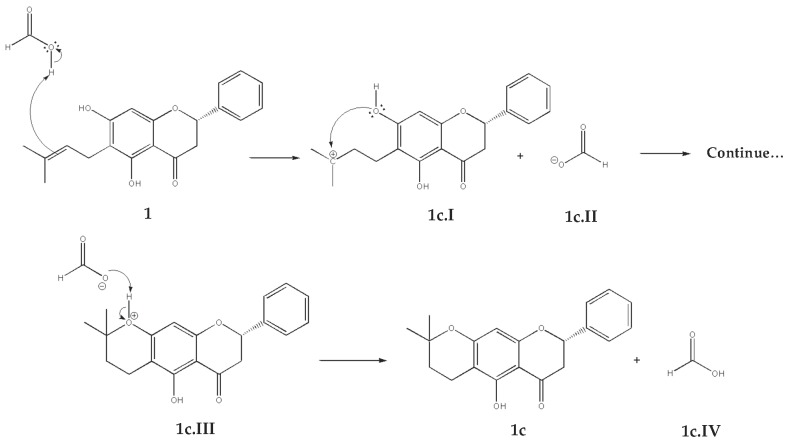
A plausible mechanism for semi-synthesis of (8*S*)-5-hydroxy-2,2-dimethyl-8-prenyl-3,4,7,8-tetrahydro-2*H*,6*H*-Benzo[1,2-*b*:5,4-*b*′]dipyran-6-one (**1c**).

As the plausible mechanism we propose that π electrons of the double bond of the prenyl group of flavanone **1** are excited by the effect of the temperature, reacting to abstract the proton of the formic acid leading to the formation of the tertiary carbocation of the intermediary **1c.I,** that later is neutralized by the electrons of the oxygen of the hydroxyl group at the 7-position of the intermediate **1c.I** to undergo an intramolecular cyclization producing to protonated intermediate **1c.II,** which when deprotonated by the anion-formate, leads to the preparation of compound **1c** and formic acid was recovered, as illustrated in Scheme 6.

The crude product was purified by preparative thin layer chromatography (TLC) to isolate derivate **1c** and the melting point was determined in the range 128–130 °C. This compound **1c** was a yellow solid. IR spectrum revealed the characteristic bands of lead flavanone **1** with the exception of the double bond carbon-carbon bands that appear in the natural flavanone **1** at 1642 cm^−1^, since the new compound **1c** has methylenes.

On the other hand, the ^1^H-NMR spectrum of compound **1c** showed signals for the hydrogens on the C-3 and C-4 single bond. In the case of the hydrogens at C-1 of prenyl group of flavanone **1** at 3.26 ppm they appear as a doublet, but in the case of the derivative **1c** they are seen as a doublet of doublet of doublets at 2.62 ppm, relative to the neighboring hydrogens H-3α and H-3β and the hydrogen found in the same carbon atom 4. Furthermore, the signal at 5.24 ppm was observed as a triplet for the vinylogous hydrogen of the C-3 position in prenyl group at natural flavanone **1**. However, in derivative **1c** this signal changed in the ^1^H-NMR spectrum. A triple signal at 1.79 ppm was evidenced. In order to verify the cyclized nature of the derivative the signals of methylene carbons C-3 and C-4 were obtained, which were at 32.23 ppm and 16.13 ppm in the ^13^C-NMR spectrum, respectively. These same signals could be observed in DEPT experiment as methylene carbons. The signal of C-2 was observed at 76.67 ppm, corresponding to the carbon that supports the methyl groups, which were observed at 27.05 ppm and 27.23 ppm, respectively, and correlated with the methyl groups characteristic of the prenyl moiety at natural flavanone **1**.

**Figure 2:**
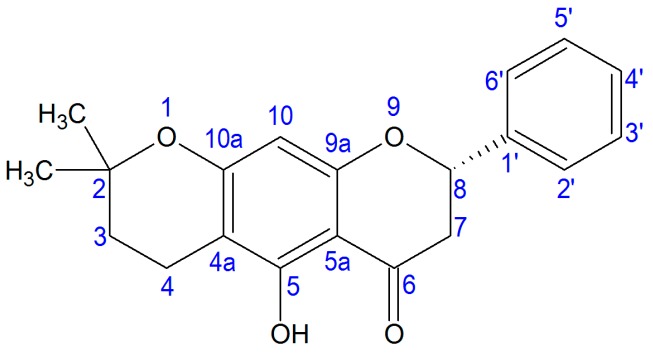
Compound **1c** and its assigned numbers.

Derivative **1c** (Figure 2) is a novel compound, which was not previously been reported in the literature and the name was assigned as (8*S*)-5-hydroxy-2,2-dimethyl-8-prenyl-3,4,7,8-tetrahydro-2*H*,6*H*-Benzo[1,2-*b*:5,4-*b*′]dipyran-6-one according to the nomenclature reported by Ahluwalia et al. [24].

#### 2.2.4. Vinylogous-Cyclization

**Scheme 7:**
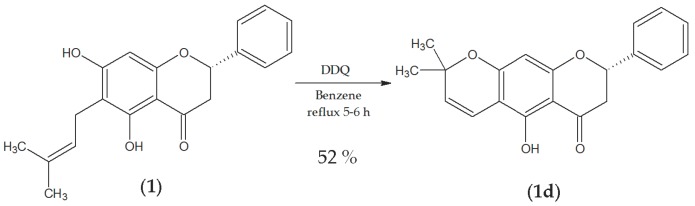
Reaction of vinylogous-cyclization of (2*S*)-5,7-dihydroxy-6-(3-methyl-2-buten-1-yl)-2-phenyl-2,3-dihydro-4*H*-1-Benzopyran-4-one (**1**) gives (8*S*)-5-hydroxy-2,2-dimethyl-8-phenyl-7,8-dihydro-2*H*,6*H*-Benzo[1,2-*b*:5,4-*b*′]dipyran-6-one (**1d**).

Vinylogous cyclized derivative **1d** (Scheme 7), was purified by recrystallization and obtained as a yellow solid with a melting point 79–83 °C. Derivative **1d** modifies the conformational freedom by forming making a new pyran ring that creates more rigid derivative and confers extra reactivity due to the π-electron contribution at carbons 3 and 4 [16].

**Scheme 8:**
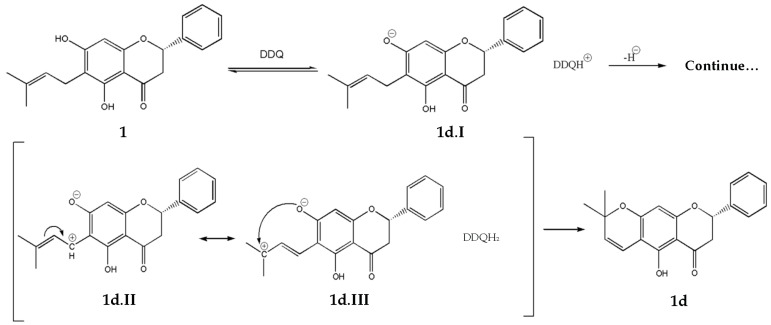
Proposed mechanism for semi-synthesis of (8*S*)-5-hydroxy-2,2-dimethyl-8-phenyl-7,8-dihydro-2*H*,6*H*-Benzo[1,2-*b*:5,4-*b*′]dipyran-6-one (**1d**).

The intramolecular cyclization of flavanone **1** to form compound **1d** by the use of 2,3-dichloro-5,6-dicyanobenzoquinone (DDQ) under anhydrous conditions is schematized in the Scheme 8. As a first step in the reaction the DDQ was protonated by abstracting the proton from the alcohol of position 7 of flavanone **1**, to produce intermediate **1d.I** and DDQH^⊕^, which aromatizes by abstraction of hydride and forms DDQH_2_ with intermediate **1d.II**. Once the intermediate **1d.II** is formed and a cationic transposition occurs generating the resonant form to obtain the more stable tertiary carbocation corresponding to intermediate **1d.III**, then the alkoxy anion carries out intramolecular cyclization by compensating the carbocation charge deficiency and thereby giving compound **1d**.

The IR spectrum of the vinylogous cyclization product **1d** showed characteristic bands of hydroxyl, ketone carbonyl groups and carbon-carbon bond of aromatic ring. The ^1^H-NMR spectrum exhibited doublet signals at 5.51 ppm and 6.62 ppm, corresponding to the vinylogous hydrogens in positions H-3 and H-4, respectively. These were different from derivative **1c**. The C=C double bond was confirmed by ^13^C-NMR signals at 126.38 ppm and 115.50 ppm for the C-3 and C-4 position carbons, respectively. The rest of the observed signals of the ^1^H-NMR and ^13^C-NMR spectra of derivatives **1c** and **1d** revealed significant changes [25]. Derivative **1d** was previously reported by Manchanda et al. in 1976 [26].

### 2.3. Cytotoxic Assay of Free Compounds

#### 2.3.1. Brine Shrimp (*Artemia salina*)

The results in Table 1 show the percentage mortality values of natural flavanone **1** and its derivatives **1a**, **1b**, **1c** and **1d** against *Artemia salina*; these values with 95% confidence intervals values were calculated by the probit analysis method [27]. It could be observed that natural flavanone **1** did not show important percentage of mortality, since at 100 ppm concentration only 11.5% nauplii mortality was detected. At the lowest concentration no toxicity was observed. However, the results allowed ascertaining changes in the biological activity due to the structural modifications applied. Derivatives **1a** and **1c** showed higher mortality rates of 36.7% and 50%, respectively, at the lowest concentration (10 ppm).

It was possible to see that the mortality rate results for **1a** and **1c** indicate the threshold of effectiveness as the effect was not modified even at increasing concentrations. Regarding derivatives **1b** and **1d**, it was possible to conclude that the structural changes resulted in a reduction of the effect of flavanone **1**, which corroborated that the cytotoxicity activity observed in flavanone **1** is related to the presence of the hydroxyl group in position 7, as well as the free disposition of the prenyl group in position 6. The implication of double bond of both the prenyl group and π-electrons of derivative **1d** was also recognized. They had not correlation since the final cytotoxicity results were different.

Therefore, as already mentioned, the change from **1** to **1b** led to the loss of pharmacological activity at least in this biological assay. In the case of **1d**, the activity may be unpredictable and the conjugated double bond may result in the generation of cytotoxic metabolites. Additionally, an antimicrobial activity has been reported for these kinds of prenylated flavonoids due to the existence of π-electrons. These compounds are more hydrophobic than the conventional flavonoids, and thus they may penetrate easily through the cell membrane [28]. Results show that all compounds have a LC_50_ > 1000 ppm (3.0820 µM).

#### 2.3.2. Cell Viability MiaPaCa-2 Cells

The MiaPaCa-2 cell line (pancreatic cancer cells) is used as an in vitro model to study pancreatic ductal adenocarcinoma carcinogenesis [29]. The results showed that derivatives **1b** and **1d** decreased cell viability in the concentration range 10–50 μM and 25–50 μM, respectively. Cell viability in these ranges of concentration was lower than that observed in flavanone **1** (Table 2). It can be hypostatized that the observed cytotoxic activity of derivatives **1b** and **1d** may be due to existence of the methyl ether group at position 7 in **1b**, and the formation of 3,4-dehydropyran ring in **1d**. Other prenylated flavanones with similar structures, such as those derived from naringenin, aromadendrin, tangeretin, among others, have been considered as potential cancer chemopreventive agents for their similar activities on cell viability at low concentrations [30,31].

A structural analysis could be performed, as well as a mechanism of action elucidation based on the bioisomeric criteria in drug design [32]. Equally, as a result of this analysis regarding different antineoplastic agents used in pancreatic cancer treatment, which have been recommended by the American Cancer Society, such as 5-fluorouracil, paclitaxel, irinotecan, *cis*-platinum and gemcitabine, a similarity in functional groups such as the aromatic hydrocarbons and amine can be observed. In the course of the analysis with derivatives providing the lowest cell viability (compounds **1b** and **1d**), these compounds possess ether groups and double bonds, as well as aromatic hydrocarbons. The first two types of groups are classical isosteres of the amino group [31], which is responsible of the decrease in cell viability against MiaPaCa-2 cells. On the other hand, in an attempt to determine the possible mechanism of action, apoptosis by similarity with anti-neoplastic drugs it could be proposed [33] as it has been demonstrated that flavonoids are potential agents in the preventive treatment of pancreatic cancer [34].

### 2.4. PLGA NPs

The use of polymeric drug delivery systems for effective encapsulation and targeting involves the right choice of polymer composition, stabilizer, solvent and elaboration technique [8]. The encapsulation of cytotoxic drugs (natural and derivatives) in particulate delivery systems represents an innovative alternative to minimize side effects, while preserving the cytotoxic activity. Among these polymers, PLGA, which was approved by the U.S. Food and Drug Administration (FDA), has been extensively used for controlled drug delivery systems because of its biocompatibility and biodegradability [35]. Poloxamer 188 (P188) was chosen as surfactant to stabilize this particulate system in aqueous suspension due to its non-ionic properties. It is also accepted by the regulatory authorities for human administration [36]. Additionally, it contributes to the stabilization of the colloidal system [37]. Based on these considerations, PLGA unloaded NPs (blank NPs), as well as flavanone (natural and derivatives)-loaded NPs were developed at a concentration of 1.5 mg/mL. The concentrations of each natural flavanone **1** and derivatives **1a**, **1b**, **1c** and **1d** loaded PLGA NPs and entrapment efficiency are expressed in Table 3.

These results show that all NPs entrapped between 78.28 and 88.47% of the compounds. The EE was high enough and ranged from 88.47% for NPs**1a** to 85.00% for NPs**1b**, these values of PLGA NPs were in agreement or even higher than other authors [38] and could be explained by the hydrophobic nature of compounds. Table 4 summarizes the obtained NPs characterization results. Characterization techniques revealed spherical shaped NPs with an overall average size (Z-average) ranging from 141.633 ± 0.773 to 205.200 ± 0.265 nm. The width of the size distribution expressed as polydispersity index (PDI) ranged between 0.058 ± 0.053 and 0.101 ± 0.031. The surface charge of NPs was obtained in terms of ZP values, which ranged from −10.633 ± 0.231 to −4.237 ± 0.242 mV.

As can be observed from Table 4, the particle size distribution was very narrow for NPs**1a**, NPs**1c** and NPs**1d**, being the highest PDI value 0.101 for NPs**1c**, which denoted the existence of a monodisperse systems. Additionally, the highest Z-average value was observed in NPs**1**. Value of ZP in PLGA NPs depends on several factors: type of PLGA, type of drug encapsulated and type of stabilizing agent used during preparation. All NPs showed negative charges ranging from −4.24 to −10.6 mV, probably due to the free carboxylic end-groups of the polymer chains in presence of water [39]. The intermolecular bonds between carbonyl group of PLGA and -OCH_3_ and -OH groups of derivative **1b** in NPs**1b** could explain its lowest Z-average and ZP values value.

**Figure 3:**
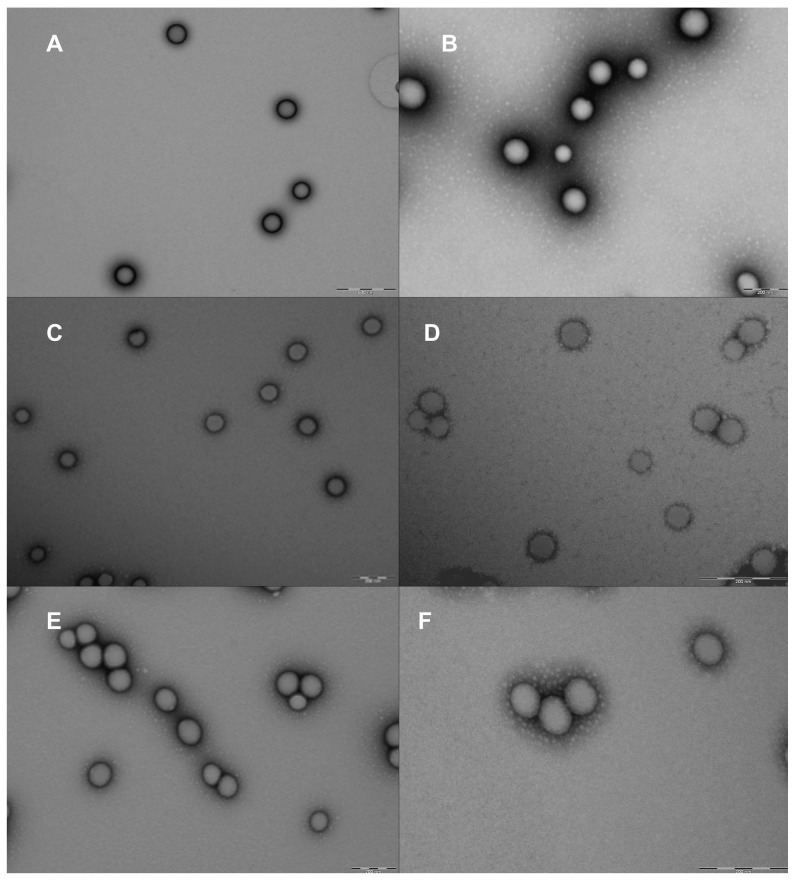
Transmission electron microscopy (TEM) images of blank NPs (**A**); and drug loaded NPs: NPs**1** (**B**); NPs**1a** (**C**); NPs**1b** (**D**); NPs**1c** (**E**) NPs**1d** (**F**). Bar lengths: 500 nm (**A**), 200 nm (**B**–**F**).

Figure 3 shows the transmission electron microscopy (TEM) images of the NPs. The morphology of NPs where most of particles had round, uniform shapes can be observed. Particle size measured by TEM was generally well correlated with that found using photon correlation spectroscopy (PCS).

Table 5 shows average NPs diameters obtained from TEM. These results were smaller than those obtained by Dynamic Light Scattering (Table 3). However, the NPs size behavior is similar, since the formulation NPs**1b** in both cases shows the smaller average NPs size, and those with larger size are blank NPs, and NPs loaded with natural flavanone (NPs**1**).

### 2.5. Cytotoxic Activity of NPs

The results of cytotoxic activity of NPs against MiaPaCa-2 cell line are listed in Table 6. Blank NPs exhibited no cytotoxicity against MiaPaCa-2 cells, and thus did not affect the results of the active substances loaded in NPs. NPs**1a** and NPs**1b** were the formulations which exhibited the lowest cell viability percentages. Therefore and taking into account the previous results of cytotoxicity of free flavanone **1a** (Table 2) it could be concluded that the encapsulation of this derivative in the NPs led to an improvement of the cytotoxic efficacy. At 50 μM concentration free flavanone **1a** reached 36.54% of cell viability whereas NPs**1a** reached 11.22%. On the other hand, NPs**1b** showed similar values of cell viability compared with free derivative **1b** at 50 μM, 31.88 ± 2.43% and 28.74 ± 2.53%. In this case, additional advantages provided by the vehicle might be expected. Finally, at 100 μM concentration values optimal values of cell viability were obtained 1.76% and 8.47% for NPs**1a** and NPs**1b**, respectively.

This improvement provided by the vehicle could be due to the different interaction mechanism between free compound and the NPs vehicle in the cellular uptake. The free compound uptake may be due to a diffusion mechanism, so after attaining saturation inside the cytoplasm further entry will be restricted and the internalized fraction inside the cell exerts the cytotoxic activity [40]. On the other hand, as is known this kind of NPs are an ideal for formulation due to its wide medical use, biocompatibility, and safety. 6-Coumarin loaded in PLGA NPs was reported in the literature and they had been shown that have multiple endocytosis internalization mechanism and to know how the mechanism action. We consider could be to realize a study the internalization of nanoparticles in cell surface [41]. Once in the cytoplasm the drug encapsulated is released from NPs as the vehicle degrades providing a sustained release exerting its cytotoxic effect [42].

**Figure 4:**
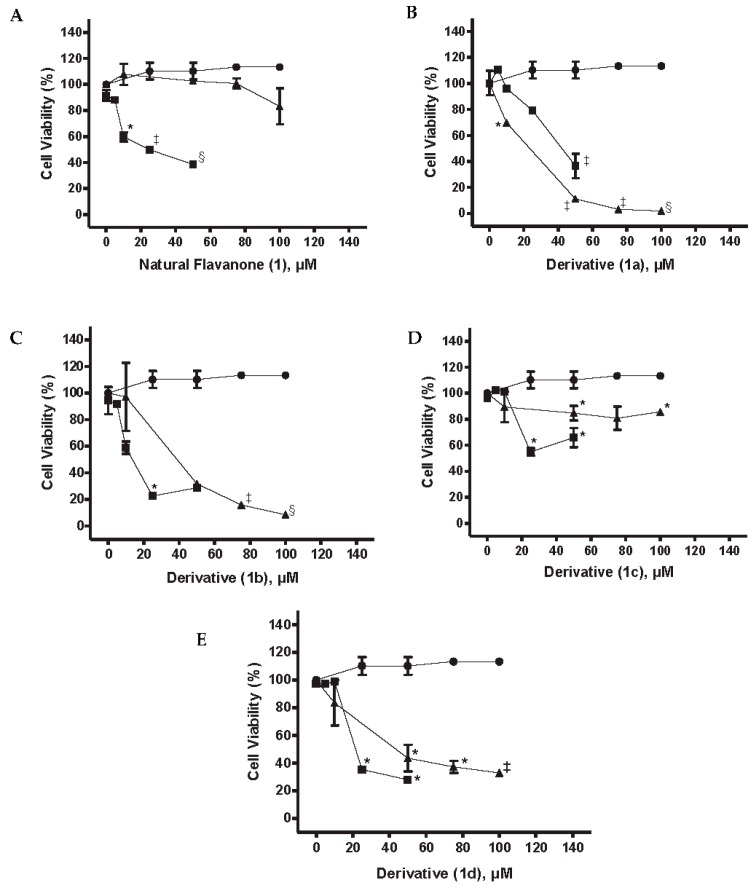
Dose dependent cytotoxicity against MiaPaCa-2 cell line of blank NPs (●), natural flavanone or derivative (■), and natural flavanone or derivative loaded in NPs (▲); natural flavanone (**1**) (**A**), derivative (**1a**) (**B**); derivative (**1b**) (**C**); derivative (**1c**) (**D**); derivative (**1d**) (**E**). Each point represents the mean ± SD (*n* = 3). (*) Denotes statistically significant differences regarding blank NPs, *p* < 0.05, (‡) denotes statistically significant differences regarding blank NPs, *p* < 0.001, and (§) denotes statistically significant differences regarding blank NPs, *p* < 0.0001.

All the studied compounds and loaded NPs exhibited a typical dose dependent cytotoxic effect represented in Figure 4. Finally, NPs**1a** was the formulation that showed the best results of cytotoxicity.

These results suggest that the compound **1b** might be a possible candidate for future investigations about cancer chemotherapy studies. The flavanone **1** and its derivatives with specified cytotoxic activity could be tested against different types of tumor cell lines. Besides, new nanostructured formulations will also be developed in the search of new therapeutics targets such as inflammation. Consequently, further studies will be conducted to investigate their possible mechanism of action.

## 3. Materials and Methods

### 3.1. Plant Material

Leaves of *E. platycarpa* Pennell & Safford (Fabaceae) were collected from the municipality of Tetipac, Guerrero State (Mexico). Voucher specimens were authenticated by Prof. Ramiro Cruz Durán (voucher specimen 1325) and were stored at the Sciences Faculty Herbarium facilities (Universidad Nacional Autónoma de México, Mexico City, Mexico).

### 3.2. Materials and Instrumentation

Solvents, deuterated solvents and reagents were all purchased from Sigma-Aldrich (Toluca de Lerdo, Mexico). Fourier transform infrared absorption spectroscopy (FTIR) measurements were performed by using a Nicolet 6700 FTIR spectrometer (Thermo Electron Scientific; Madison, WI, USA) in the range of 525–4000 cm^−1^ equipped with a KBr beam splitter, a deuterated triglycine sulfate (DTGS) detector and OMNIC^®^ software (Thermo Electron). ^1^H- and ^13^C-NMR spectra were recorded in a Unity NMR spectrometer (Varian Inova, Palo Alto, CA, USA) operating at 400 MHz for ^1^H and 200 MHz for ^13^C nuclei. Fifteen mg of each compound were prepared in deuterated chloroform (CDCl_3_) and tetramethylsilane (TMS) was used as internal standard. The molecular formulae of compounds **1a**, **1b**, **1c** and **1d** was confirmed by FABSMS analysis performed on a JMX-AX 505 HA mass spectrophotometer (JEOL Ltd., Tokyo, Japan).

### 3.3. Preparation of Methanolic Extract

The methanolic extract of *E. platycarpa* leaves was obtained by maceration method. Leaves were dried at room temperature in the shade. Once the leaves were dried they were pulverized and extracted with methanol by maceration at room temperature three times (100 g of dried vegetable material per 1000 mL of methanol). Then, the extractions were performed and the solvent was removed under reduced pressure to obtain the corresponding residues [14].

### 3.4. Isolation of Compound (2S)-5,7-Dihydroxy-6-(3-methyl-2-buten-1-yl)-2-phenyl-2,3-dihydro-4H-1-benzopyran-4-one *(**1**)*

From the methanolic extract of *E. platycarpa* leaves the prenylated flavanone compound **1** (329 mg), was isolated as yellow solid by column chromatography at reduced pressure using silica gel HF 254. Then the flavanone was purified and characterized by direct TLC comparison with an original sample available at the laboratory. It was also analyzed by ^1^H-NMR (400 MHz) and ^13^C-NMR, as well as mass spectrometry checking their identities by comparison with the previously published spectroscopic data [14].

Compound **1** can also be found (CAS No.: 55051-77-9).

### 3.5. Preparation of Derivatives

(2*S*)-5,7-Bis(acetyloxy)-6-(3-methyl-2-buten-1-yl)-2-phenyl-2,3-dihydro-4*H*-1-Benzopyran-4-one (**1a**). Compound **1** (50.00 mg 0.1541 mmol) in acetone (2 mL) was treated with a (2:1) mixture of Ac_2_O:Py (4 mL) and room temperature for 24 h. After usual work-up 39.8 mg of a residue was obtained, which was purified by TLC (eluting with 9:1 *n*-hexane:EtOAc) to give compound **1a**, as a yellow solid in 79.6% yield. Melting point 72–78 °C. ^1^H-NMR (400 MHz, chloroform-*d*, δ; ppm) 7.43 (m, 5H, 2-C_6_H_5_), 6.78 (s, 1H, 8-H), 5.46 (dd, *J* = 12.8, 3.2 Hz, 1H, 2-H), 5.00 (tc, *J* = 6.8, 1.2 Hz, 1H, 2-H, prenyl moety), 3.18 (sa, 1H, 1-H, prenyl moety), 3.06 (dd, *J* = 17.2, 12.8 Hz, 1H, 3-H), 2.79 (dd, *J* = 17.2, 3.2 Hz, 1H, 3-H), 2.43 (s, 3H, 5-OCOCH_3_), 2.32 (s, 3H, 7-OCOCH_3_), 1.75 (s, 3H, 3-CH_3_, prenyl moety), 1.70 (s, 3H, 4-H, prenyl moety); ^13^C-NMR (200 MHz, Chloroform-*d*, δ; ppm) 18.26 (q, 3-CH_3_, prenyl moety); 21.32 (q, 7-OCOCH_3_); 21.44 (q, 5-OCOCH_3_).; 23.35 (t, 1-C, prenyl moety); 25.97 (q, 4-C, prenyl moety); 45.67 (t, 3-C); 79.63 (d, 2-C); 110.13 (d, 8-C); 111.97 (s, 4a-C); 120.99 (d, 2-C, prenyl moety); 120.99 (s, 6-C); 126.16 (d, 2′-C/6′-C); 128.83 (d, 3′-C/5′-C); 128.86 (d, 4′-C); 132.36 (s, 3-C, prenyl moety); 138.18 (s, 1′-C); 149.11 (s, 5-C); 154.66 (s, 7-C); 160.79 (s, 8a-C); 168.02 (s, 5-OCOCH_3_); 169.05 (s, 7-OCOCH_3_); 189.20 (s, 4-C). IR; 2921.8 cm^−1^, 2371.9 cm^−1^, 1770.6 cm^−1^, 1688.7 cm^−1^, 1608.0 cm^−1^, 1451.5 cm^−1^, 1370.5 cm^−1^, 1283.0 cm^−1^, 1193.5 cm^−1^, 1155.3 cm^−1^, 1107.0 cm^−1^, 1005.9 cm^−1^, 895.9 cm^−1^, 764.6 cm^−1^, 699.3 cm^−1^, 577.8 cm^−1^. HR-MS (ESI), calcd. for C_24_H_24_O_6_: [M + H]^+^ 409.1573, found: 409.1853 ([M + H]^+^).

(2*S*)-5-Hydroxy-7-methoxy-6-(3-mehyl-2-buten-1-yl)-2-phenyl-2,3-dihydro-4*H*-1-Benzopyran-4-one (**1b**). A solution of flavanone **1** (50.00 mg; 0.1541 mmol) in a mixture of absolute ethanol and ethyl ether (1:1) was treated with an ethereal solution of diazomethane prepared from nitromethyl urea (2.8 g). The methylation agent was added in small lots and cooled in ice for 10 min. The color of the reaction changed to pale yellow and the reaction mixture was then kept in an ice-chest for 5 h. The container was closed with a cork with a thin tube to enable any gases released to escape. The solvents were completely distilled off and the residue taken up in a vial. After the solution was concentrated and cooled in ice-water for a few hours the methyl ether crystallized. It was purified by recrystallization to give a yellow solid in 58% yield (29.0 mg). Melting point 95–96 °C. ^1^H-NMR (400 MHz, chloroform-*d*, δ; ppm) 12.05 (s, 1H, 5-OH); 7.43 (m, 2-C_6_H_5_); 6.09 (s, 1H, 8-H); 5.40 (dd, *J* = 13.2, 3.2 Hz, 1H, 2-H); 5.18 (t, *J* = 7 Hz, 1H, 2-H, prenyl moety); 3.83 (s, 3H, 7-OCH_3_); 3.26 (d, *J* = 7 Hz, 1H, 1-H, prenyl moety); 3.08 (dd, *J* = 17.2, 13.2 Hz, 1H, 3a-H); 2.79 (dd, *J* = 17.2, 3.2 Hz, 1H, 3b-H); 1.77 (s, 3H, 3-CH_3_, prenyl moety); 1.67 (s, 3H, 4-H, prenyl moety). ^13^C-NMR (200 MHz, Chloroform-*d*, δ; ppm) 18.08 (q, 3-CH_3_, prenyl moety); 21.37 (t, 1-C, prenyl moety); 26.15 (q, 4-C, prenyl moety); 43.79 (t, 3-C); 56.14 (q, 7-OCH_3_); 79.62 (d, 2-C); 91.24 (s, 8-C); 103.16 (s, 4a-C); 110.29 (d, 6-C); 122.46 (d, 2-C, prenyl moety); 126.35 (d, 4′-C); 126.35 (d, 2′-C/6′-C); 129.082 (d, 3′-C/5′-C); 131.91 (s, 3-C, prenyl moety); 138.68 (s, 1′-C); 160.42 (s, 5-C); 161.51 (s, 8a-C); 165.66 (s, 7-C); 195.92 (s, 4-C). IR; 3449 cm^−1^, 2918.9 cm^−1^, 1717.6 cm^−1^, 1647.5 cm^−1^, 1612.2 cm^−1^, 1485.3 cm^−1^, 1445.6 cm^−1^, 1358.3 cm^−1^, 1294.7 cm^−1^, 1204.9 cm^−1^, 1157.1 cm^−1^, 1099.3 cm^−1^, 995.5 cm^−1^, 901.6 cm^−1^, 810.8 cm^−1^, 742.5 cm^−1^, 696.2 cm^−1^, 632.4 cm^−1^, 554.7 cm^−1^, 445.7 cm^−1^. HR-MS (ESI), calcd. for C_21_H_22_O_4_: [M + H]^+^ 339.1518, found: 339.1604 ([M + H]^+^). It was also compared with data available in literature [21]. Compound **1b** can also be found (CAS No.: 55051-79-1).

(8*S*)-5-Hydroxy-2,2-dimethyl-8-prenyl-3,4,7,8-tetrahydro-2*H*,6*H*-Benzo[1,2-*b*:5,4-*b*′]dipyran-6-one (**1c**). A formic acid solution (20 mL) containing **1** (50.00 mg, 0.1541 mmol) was heated under reflux up to 60 °C for 1.5 h, and then it was left to cool for 6 h at room temperature. The crude of the reaction product was extracted by ethyl acetate (EtOAc). Later, it was washed 3 times with water and the dampness was eliminated with anhydrous Na_2_SO_4_. The compound **1c** was obtained as a yellow solid in 87.2% yield (43.6 mg), after removal of the solvent. Melting point 128–130 °C [43]. ^1^H-NMR (400 MHz, chloroform-*d*, δ; ppm) 12.39 (s, 1H, 5-OH); 7.44 (m, 5H, 8-C_6_H_5_); 5.95 (s, 1H, 10-H); 5.38 (dd, *J* = 12.8, 3.2 Hz, 1H, 8-H); 3.06 (dd, *J* = 17.2, 12.8 Hz, 1H, 7-Hα); 2.80 (dd, *J* = 17.2, 3.2 Hz, 1H, 7-Hβ); 2.62 (ddd, *J* = 6.8, 2 Hz, 2H, 4-H); 1.79 (t, *J* = 6.8 Hz, 2H, 3-H); 1.34 (s, 6H, 2-CH_3_). ^13^C-NMR (200 MHz, chloroform-*d*, δ; ppm) 16.13 (t, 4-C); 27.05 (q, 2-CH_3_); 27.23 (q, 2-CH_3_); 32.23 (t, 3-C); 43.86 (t, 7-C); 76.67 (s, 2-C); 79.12 (d, 8-C); 96.43 (d, 10-C); 102.30 (s, 4a-C); 102.42 (s, 5a-C); 126.16 (d, 2′-C/6′-C); 128.72 (d, 4′-C); 128.82 (d, 3′-C/5′-C); 138.73 (s, 1′-C); 160.21 (s, 5-C); 161.28 (s, 10a-C); 162.82 (s, 9a-C); 195.50 (s, 6-C). IR; 3753 cm^−1^, 3448 cm^−1^, 2921 cm^−1^, 2371 cm^−1^, 1637 cm^−1^, 1447 cm^−1^, 1376 cm^−1^, 1305 cm^−1^, 1168 cm^−1^, 1119 cm^−1^, 899 cm^−1^, 805 cm^−1^, 758 cm^−1^, 694 cm^−1^, 607 cm^−1^, 479 cm^−1^. HR-MS (ESI), calcd. for C_20_H_20_O_4_: [M + H]^+^ 325.1362, found: 325.1403 ([M + H]^+^).

(8*S*)-5-Hydroxy-2,2-dimethyl-8-phenyl-7,8-dihydro-2*H*,6*H*-Benzo[1,2-*b*:5,4-*b*′]dipyran-6-one (**1d**). Flavanone **1** (50.00 mg, 0.1541 mmol) was treated with 2,3-dichloro-5,6-dicyanobenzoguinone (DDQ, 40.00 mg) in equimolar proportions (1:4) under reflux up to 78 °C in sodium-dried benzene (10 mL) for 5 h . The crude reaction product (40 mg) was submitted to TLC eluted with a 3:7 hexanes-CH_2_Cl_2_ mixture to obtain the compound **1d** [25] in 52% yield (26 mg). ^1^H-NMR (400 MHz, chloroform-*d*, δ; ppm) 12.30 (s, 5-OH); 7.45 (m, 5H, 8-C_6_H_5_); 6.62 (d, *J* = 10 Hz, 1H, H-4); 5.97 (s, 1H, H-10); 5.51 (d, *J* = 10 Hz, 1H, H-3); 5.40 (dd, *J* = 13.2, 3.2 Hz, 1H, H-8); 3.10 (dd, *J* = 17.2, 13.2 Hz, 1H, H-7α); 2.81 (dd, *J* = 17.2, 3.2 Hz, 1H, H-7β); 1.44 (s, 3H, 2-CH_3_); 1.43 (s, 3H, 2-CH_3_). ^13^C-NMR (200 MHz, chloroform-*d*, δ; ppm) 28.56 (q, 2-CH_3_); 28.64 (q, 2-CH_3_).43.59 (t, 7-C); 78.52 (s, 2-C); 79.31 (d, 8-C); 96.50 (d, 10-C); 103.07 (s, 5a-C); 103.30 (s, 4a-C); 115.50 (d, 4-C); 126.38 (d, 3-C); 126.50 (d, 2′-C/6′-C); 128.39 (d, 4′-C); 129.054 (d, 3′-C/5′-C); 138.648 (s, 1′-C); 158.66 (s, 5-C); 162.53 (s, 10a-C); 162.37 (s, 9a-C); 195.97 (s, 6-C); IR; 3433 cm^−1^, 2927 cm^−1^, 2871 cm^−1^, 2366 cm^−1^, 1735 cm^−1^, 1637 cm^−1^, 1458 cm^−1^, 1372 cm^−1^, 1292 cm^−1^, 1166 cm^−1^, 1124 cm^−1^, 981 cm^−1^, 901 cm^−1^, 762 cm^−1^, 695 cm^−1^, 480 cm^−1^. HR-MS (ESI), calcd. for C_20_H_18_O_4_: [M + H]^+^ 323.1267, found: 323.1267 ([M + H]^+^). Compound **1d** can also be found (CAS No.: 882846-01-7).

### 3.6. Preparations PLGA NPs

NPs loaded with natural flavanone **1** and derivatives **1a**, **1b**, **1c** and **1d** were prepared by the solvent displacement technique reported by Fessi et al. [44]. Briefly, a 50:50 organic solution of PLGA (90 mg) in acetone (25 mL) containing the drug (1.0 mg/mL) was poured under moderate stirring into P188 aqueous solution (10 mL, 10 mg/mL, pH = 3.5). The acetone was then evaporated and the volume of NP dispersion was concentrated under reduced pressure on a B-480 rotary evaporator (Büchi, Labortechnik AG, Flawil, Switzerland) [8]. Once the organic solvent was removed the obtained NPs were cleaned using repeated cycles of centrifugation and resuspension in double distilled water.

#### 3.6.1. Particle Size Analysis

The size of NPs was determined by PCS by using a Zetasizer Nano ZS (Malvern Instruments, Malvern, UK), a non-invasive, well established technique for measuring the size of particles in the submicron region, providing in parallel the PDI. Samples were directly placed into the module and the data were collected at room temperature.

#### 3.6.2. Zeta Potential Measurements

The ZP of NPs was measured using a Zetasizer Nano ZS (Malvern Instruments, Malvern, UK). This instrument also allows determining the electrophoretic mobility to assess the surface electrical charge of particles. Samples were diluted in purified water adjusting conductivity (50 µS/cm) with sodium chloride solution (0.9% *w*/*v*) in order to avoid fluctuations of the ZP caused by differences in the conductivity of distilled water [45]. The ZP was calculated from the electrophoretic mobility using the Helmholtz—Smoluchowski equation [46].

#### 3.6.3. Morphological Studies

Morphological examination of polymeric NPs was performed by TEM with a JEM-1010 microscope (JEOL Ltd., Tokyo, Japan). Briefly, one drop of each sample was deposited on copper grids covered with a layer of Formvar^®^ standing for 4 min. The grids were then stained with one drop of 2% uranyl acetate solution and allowed to dry for 5 min before examination. Image analysis was performed using the ImageJ 1.46r analysis software (Wayne Rasband, National Institutes of Health (NIH), Rockville, MD, USA) [47]. For this task 100 images of each NPs type were analyzed.

#### 3.6.4. Entrapment Efficiency (EE %)

The entrapment efficiency (EE) of each compound **1**, **1a**, **1b**, **1c** and **1d** loaded polymeric NPs was determined by measuring the concentration of free drug in the dispersion medium. The non-entrapped compound **1**, **1a**, **1b**, **1c** and **1d** was separated by filtration/centrifugation technique using Amicon centrifugal filter devices equipped with 100 KDa Ultracel membrane (Millipore Corporation, Billerica, MA, USA) at 3000 rpm for 12 min on a Sigma 301K centrifuge (Sigma, Barcelona, Spain). Prior to filtration/centrifugation each sample was diluted (1:20) with ethanol/water (70:30) solution to avoid deposition of free compounds (possibly crystallized in the aqueous phase) onto NP surface avoiding erroneous overestimation of the EE. NPs were then retained on the membrane filter while the hydro-ethanolic solution containing the free compound crossed the membrane. The amount of compound in this solution was established using HPLC analysis. The natural flavanone **1** and derivatives **1a**, **1b**, **1c** and **1d** entrapment efficiency (EE %) was calculated as indicated in the formula below:

(1) $$\mathrm{EE}\;\%=\frac{\mathrm{Total}\;\mathrm{amount}\;\mathrm{of}\;\mathrm{compound}\;-\;\mathrm{Free}\;\mathrm{amount}\;\mathrm{of}\;\mathrm{compound}}{\mathrm{Total}\;\mathrm{amount}\;\mathrm{of}\;\mathrm{compound}}\times 100$$

### 3.7. Cytotoxic Assays

#### 3.7.1. Brine Shrimp, *Artemia salina* Assay

The shrimp lethality assay was performed as previously reported by Mayer et al. [48]. It is based on the ability to kill laboratory cultured *Artemia salina* brine shrimp nauplii. The assay is considered a useful tool for preliminary assessment of toxicity. It has also been used for the detection of fungal toxins, plant extract toxicity, heavy metals, cyanobacteria toxins, pesticides, and cytotoxicity testing of dental materials.

Dried cysts were incubated (1 g/L) in seawater at 28–30 °C with strong aeration, under a continuous light regime. Approximately 12 h after hatching, the phototropic nauplii were collected with a pipette and concentrated in a small vial. Ten brine shrimp were transferred to each well using adequate pipette. Toxicity was determined after 24 h of exposure. The numbers of survivors were counted and the percentages of dead ones were calculated. Larvae were considered dead if they did not exhibit any internal or external movement during several seconds of observation. Percentage mortality values with 95% confidence intervals values were calculated by the probit analysis method [27,49]. Natural flavanone **1** and derivatives **1**, **1b**, **1c** and **1d** were tested at concentration level of 1000, 100 and 10 ppm. All sample stock solutions were prepared in saline solution with 0.1% dimethylsulfoxide.

#### 3.7.2. Cell Culture

MiaPaCa-2 pancreatic cancer cell lines were used throughout the study. Cells were grown in F-12 medium (Gibco, Grand Island, NY, USA) supplemented with 5% (*v*/*v*) fetal bovine serum (Gibco), 100 U/mL sodium penicillin G and 100 μg/mL streptomycin, and were maintained at 37 °C in a humidified atmosphere containing 5% CO_2_. The compounds used in cell incubations were dissolved in dimethylsulfoxide (DMSO) and the final concentration of DMSO in the medium was always kept lower than 1% (*v*/*v*) [50].

#### 3.7.3. Cell Viability Studies

Thirty thousand MiaPaca-2 cells were seeded in 35 mm diameter dishes with 2 mL of F-12 medium. Cells were cultured for 2 h without treatment and then incubated with different compounds at the indicated concentrations. After 7 days of incubation, cell growth was determined by the MTT test.

Briefly, 200 µL of a 0.5 mg/mL MTT solution [3-(4,5-dimethylthiazolyl-2)-2,5-diphenyltetrazolium bromide] (Sigma-Aldrich, Barcelona, Spain) and 700 µL of a 50 mM succinic acid solution, both in PBS, were added to each well. The plates were incubated at 37 °C for 3 h to allow the formation of formazan crystals. Then, the dark blue crystals were dissolved with 10% sodium dodecyl sulfate (SDS) in DMSO solution and their absorbance was read at 570 nm in a spectrophotometer. Results are expressed as a percentage of viability with respect to the control cells grown in the absence of compounds [51].

### 3.8. Data Analysis

All experiments were carried out in triplicate. The results were analyzed and expressed as mean ± standard deviation (SD). Statistical analysis was done using one-way analysis of variance.

## 4. Conclusions

Four derivatives **1a**, **1b**, **1c** and **1d** were prepared from natural flavanone **1** by a molecular design strategy (pharmacomodulation); two if these compounds (**1a** and **1c**) are novel compounds, and they have not been reported yet in the literature. All compounds belong to the group of flavonoids with potential importance in human health. Prenylated flavanones have an important role as potential chemotherapeutic agents. The development of semi-synthetic methodology towards the novel pyrano system of prenyl flavanones will allow the investigation of related natural productbased heterocycles of this kind of compounds. Particularly, derivative **1b** can be proposed applications. For this task biopharmaceutical studies should be accomplished.

PLGA NPs were prepared using the solvent displacement technique. The morphology, Z-average and ZP characterization demonstrated that PLGA NPs were within acceptable parameters, showing small, homogeneous and negatively charged NPs. Concretely, NPs loading derivatives **1a** and **1b** were the nano-structured formulation showing the best results against MiaPaca-2 cell line, probably due to the increment of the lipophilicity provided by an acetyl moiety and methyl group, respectively. These might also be proposed as candidates for additional research to investigate their potential medical applications.

## Figures and Tables

**Table 1:** Percentage mortality values of natural flavanone **1** and derivatives **1a**, **1b**, **1c** and **1d** against *Artemia salina*.

Compounds	Concentration (ppm/µM)	Percentage Mortality Values (%)
**1**	0/0	0.0
	10/0.03083	0.0
	100/0.3083	11.5
	1000/3.0828	43.2
**1a**	0/0	0.0
	10/0.0245	36.7
	100/0.245	40.0
	1000/2.45	30.0
**1b**	0/0	0.0
	10/0.02955	10.0
	100/0.29551	0.0
	1000/2.9551	0.0
**1c**	0/0	0.0
	10/0.03083	50.0
	100/0.3083	45.0
	1000/3.0828	40.0
**1d**	0/0	0.0
	10/0.03102	13.3
	100/0.3102	0.0
	1000/3.102	0.0

**Table 2:** Percentage cell viability (%) of MiaPaCa-2 of natural flavanone **1** and derivatives **1a**, **1b**, **1c** and **1d**. Concentration values are in µM, and cell viability are expressed as mean ± SD. All data were obtained from three replicates.

Concentration	Percentage Cell Viability (%) of MiaPaCa-2				
	1	1a	1b	1c	1d
DMSO and Control	91.35 ± 4.18	100.28 ± 9.39	94.27 ± 10.30	95.99 ± 1.14	97.47 ± 2.22
5 μM	88.08 ± 1.99	110.67 ± 2.03	91.65 ± 2.04	102.18 ± 1.05	97.24 ± 1.20
10 μM	59.63 ± 3.54	96.19 ± 1.40	58.94 ± 4.58	101.23 ± 1.45	98.81 ± 1.70
25 μM	49.80 ± 1.19	79.22 ± 1.16	22.56 ± 1.69	54.82 ± 2.84	35.35 ± 2.50
50 μM	38.48 ± 1.52	36.54 ± 9.34	28.74 ± 2.53	65.85 ± 7.46	27.86 ± 1.25

**Table 3:** Compound concentrations (mM) loaded in PLGA NPs and entrapment efficiency (EE %).

PLGA Nanoparticles	Concentration (mM)	Entrapment Efficiency (EE %)
NPs**1**	4.62	80.00 ± 4.75
NPs**1a**	3.67	88.47 ± 4.18
NPs**1b**	4.43	85.00 ± 5.80
NPs**1c**	4.62	78.28 ± 5.85
NPs**1d**	4.65	78.75 ± 4.34

**Table 4:** Z-Average (nm), polydispersity index and zeta potential (mV) of developed NPs. All data were obtained from three replicates and values are expressed as the mean ± SD.

Nanoparticles	Z-Average (nm)	Polydispersity Index	Zeta Potential (mV)
Blank	195.667 ± 1.069	0.054 ± 0.016	−4.237 ± 0.242
NPs**1**	205.200 ± 0.265	0.058 ± 0.053	−8.250 ± 0.346
NPs**1a**	178.033 ± 1.332	0.080 ± 0.004	−9.053 ± 0.323
NPs**1b**	141.633 ± 0.777	0.086 ± 0.017	−10.633 ± 0.231
NPs**1c**	175.167 ± 0.603	0.101 ± 0.031	−6.480 ± 0.377
NPs**1d**	173.400 ± 1.587	0.064 ± 0.007	−6.647 ± 0.405

**Table 5:** Average diameter of NPs (nm) obtained by image analysis software (ImageJ 6.0) from TEM. All data were obtained from 100 particles and values are expressed as mean ± SD.

Nanoparticles	Z-Average (nm)
Blank	157.295 ± 10.405
NPs**1**	112.779 ± 19.232
NPs**1a**	76.430 ± 7.197
NPs**1b**	54.351 ± 7.156
NPs**1c**	102.228 ± 11.656
NPs**1d**	81.933 ± 8.500

**Table 6:** Percentage cell viability (%) of MiaPaCa-2 against developed NPs. Concentration values are in µM for NPs, and cell viability is expressed as mean ± SD. All values were obtained from three replicates.

Concentration	Percentage Cell Viability (%) of MiaPaCa-2 against Developed NPs					
	Blank NPs	NPs1	NPs1a	NPs1b	NPs1c	NPs1d
0 μM	100 ± 0.00	100 ± 0.00	100 ± 0.00	100 ± 0.00	100 ± 0.00	100 ± 0.00
10 μM	111.24 ± 6.44	107.75 ± 8.18	69.62 ± 2.26	97.12 ± 25.64	89.44 ± 11.74	83.51 ± 16.33
50 μM	111.24 ± 6.44	102.68 ± 1.64	11.22 ± 2.36	31.88 ± 2.43	84.66 ± 5.60	43.59 ± 9.60
75 μM	113.40 ± 15.50	100.75 ± 4.00	3.20 ± 1.88	15.88 ± 2.40	80.68 ± 9.03	37.30 ± 4.33
100 μM	113.40 ± 15.50	83.24 ± 13.81	1.76 ± 1.01	8.47 ± 1.05	85.69 ± 2.41	32.85 ± 2.01
